# A New Neurorehabilitative Postsurgery Intervention for Facial Palsy Based on Smile Observation and Hand-Mouth Motor Synergies

**DOI:** 10.1155/2021/8890541

**Published:** 2021-03-24

**Authors:** Elisa De Stefani, Anna Barbot, Chiara Bertolini, Mauro Belluardo, Gioacchino Garofalo, Nicola Bruno, Bernardo Bianchi, Andrea Ferri, Pier Francesco Ferrari

**Affiliations:** ^1^Unit of Neuroscience, Department of Medicine and Surgery, University of Parma, Italy; ^2^Unit of Audiology and Pediatric Otorhinolaryngology, University Hospital of Parma, Italy; ^3^Department of Humanities, Social Sciences and Cultural Industries, University of Parma, Italy; ^4^Maxillo-Facial Surgery Operative Unit, Head and Neck Department, University of Parma, Italy; ^5^Institut des Sciences Cognitives Marc Jeannerod, CNRS, Université de Lyon, Bron, France

## Abstract

**Objective:**

To perform a preliminary test of a new rehabilitation treatment (FIT-SAT), based on mirror mechanisms, for gracile muscles after smile surgery.

**Method:**

A pre- and postsurgery longitudinal design was adopted to study the efficacy of FIT-SAT. Four patients with bilateral facial nerve paralysis (Moebius syndrome) were included. They underwent two surgeries with free muscle transfers, one year apart from each other. The side of the face first operated on was rehabilitated with the traditional treatment, while the second side was rehabilitated with FIT-SAT. The FIT-SAT treatment includes video clips of an actor performing a unilateral or a bilateral smile to be imitated (FIT condition). In addition to this, while smiling, the participants close their hand in order to exploit the overlapped cortical motor representation of the hand and the mouth, which may facilitate the synergistic activity of the two effectors during the early phases of recruitment of the transplanted muscles (SAT). The treatment was also aimed at avoiding undesired movements such as teeth grinding. *Discussion*. Results support FIT-SAT as a viable alternative for smile rehabilitation after free muscle transfer. We propose that the treatment potentiates the effect of smile observation by activating the same neural structures responsible for the execution of the smile and therefore by facilitating its production. Closing of the hand induces cortical recruitment of hand motor neurons, recruiting the transplanted muscles, and reducing the risk of associating other unwanted movements such as teeth clenching to the smile movements.

## 1. Introduction

Moebius syndrome (MBS) is a rare neurological disorder characterized by bilateral nonprogressive congenital palsy of the facial (VII cranial) and abducens (VI cranial) nerves. Researchers estimate that the condition affects 1 in 50,000 to 1 in 500,000 newborns worldwide [1, 2]. In Italy, it is estimated that 5-6 individuals are born with MBS every year, yielding a total of about 500-600 affected patients [3]. Patients with MBS present facial and ocular symptoms at birth including reduced or absent facial expressiveness, incomplete eye closure, inability to perform lateral eye movements, and difficulty in sucking. Patients with MBS cannot perform movements such as closing their lips, pronouncing some language sounds, smiling symmetrically, closing their eyelids, or wiggling their eyebrows. They also present labial incompetence (i.e., drooling due to inability to effectively contain saliva) and difficulties in closing the eyelids, which may cause corneal ulcers or infections [4]. Other cranial nerves such as the glossopharyngeal and spinal accessory may be involved, and patients may also present limb abnormalities (i.e., clubbed feet, congenital hand anomalies, and pectoral anomalies) in up to 15%-25% of cases [5]. Most importantly, the absence of facial mimicry hinders nonverbal communication, interfering greatly with social interactions and leading to psychological repercussions such as social stigma, marginalization, and depression [1, 6].

To date, the only available treatment to partially overcome facial palsy in MBS is surgical. Facial paralysis reconstruction (i.e., smile surgery) is aimed at achieving symmetry at rest and during dynamic facial movements, thus creating some degree of mobility in the lower face to produce facial expressions [7]. Depending on the origin of the facial palsy and on its evolution over time, patients may require a muscle transfer (free functional muscle transfer, FFMT) [3]. For patients with bilateral paralysis such as MBS, FFMT is the standard procedure aimed at restoring facial animation [8, 9] (for further details on FFMT, see supplementary online material). Rehabilitation requires a prolonged period after surgery, with the patient spending many months exercising facial movements under the guidance of a speech therapist [10]. At present, no consensus guidelines for the rehabilitative protocol are available for such forms of facial palsy. Nevertheless, once the muscle begins to show evidence of producing the first contractions, clinicians have found it effective to train patients to produce muscle contractions through a teeth clenching trigger under mirror feedback [11]. Although teeth clenching has proved effective in rapidly recruiting the transplanted muscles [12], clinicians also report difficulties in dissociating the movements of muscles for chewing from those of smiling. Therefore, long periods of rehabilitation are required before patients learn to move facial muscles independently and to dissociate the motor circuits involved in chewing and smiling [13]. Moreover, some patients report discomfort in observing their image reflected in a mirror, resulting in poor compliance during home training. Indeed, it is well known that facial palsy has negative consequences for self-perception [14, 15] due to facial asymmetry and absence of facial mimicry.

The purpose of the present study was to evaluate the feasibility of a new rehabilitation treatment after smile surgery. We propose a treatment based on action observation therapy (AOT) [16], which has been shown to have clinical and rehabilitative relevance [17–20], and which exploits the visuomotor coupling properties of the mirror neuron system (MNS) [21] as well as the motor synergies between the hand and the mouth present at a cortical level [22–26] to facilitate the recruitment of transplanted muscles in MBS patients.

### 1.1. Theoretical Assumptions of Facial Imitation Treatment (FIT)

Mirror neurons were discovered in the ventral premotor region F5 of the macaque monkey more than twenty-five years ago [27, 28]. This class of neurons fires both when individuals execute a specific motor act and when they observe the same or a similar act performed by another individual [29–31]. The mirror mechanism is widely believed to support social cognitive functions such as action and emotion understanding by mapping perceived actions onto internal motor representations [32, 33]. Evidence suggests that mirror neurons are recruited in tasks requiring observation and imitation of actions and facial expressions [30, 33–36], empathy [37–40], and intentions [23, 41] and in language perception [42, 43]. These properties of mirror mechanisms can be exploited in neurorehabilitative treatments. For instance, in patients with motor deficits due to vascular brain injury or other neurological insults, the observation of a movement might improve movement recovery, reinforcing the activation of motor circuits which have been weakened due to the lesion [6, 17, 44]. This mechanism is the basis of AOT which combines exercises aimed at reducing the motor deficit with rehabilitation sessions whereby patients simultaneously observe the same exercises performed by the rehabilitator [16, 18].

**Figure 1:**
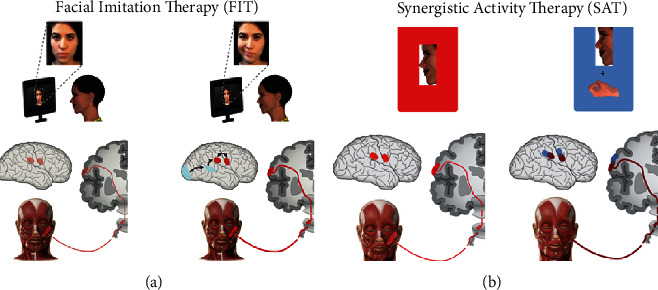
Modified from Ferrari et al. [21]: FIT-SAT theoretical assumptions. (a) FIT combined action observation with the direct effects of action execution suggesting that activation of motor areas by action observation becomes reinforced by the concomitant active execution of the observed actions^19^; (b) the synergistic activity of hand closing while smiling should facilitate the activation of the cortical areas connected to the mouth. We hypothesized that hand contraction would facilitate the recruitment of the gracilis muscle as a consequence of the activity of mouth motor neurons in motor cortical areas.

In this study, we applied the principles underlying AOT to smile rehabilitation. According to embodiment theories [18, 30, 32, 43, 45], during the observation of emotional faces, affective and motor neural systems are activated together [1, 46–48] and people would react with congruent muscle activations (unconscious facial mimicry [49]) when looking at emotional facial expressions. This covert motor simulation of emotional faces [50] is supported by a broad network of regions with mirroring properties [49] that reflect an internal simulation of the perceived emotional expression. Consequently, perceiving another person displaying a facial expression would result in increased neural activity in the perceiver's motor, emotional, and somatosensory areas [49, 51]. Thus, we hypothesized that by observing an actor who is smiling, the neural circuits that control the smile in the MBS patient may facilitate the recruitment of the transplanted muscle (Figure 1 [21]).

### 1.2. Synergistic Activity Treatment (SAT): Theoretical Assumptions

The concept of synergy has been proposed to explain the functional modules that control hand shaping while an individual is grasping objects of different sizes. Classic somatotopic theories postulate that distinct clusters of neuronal populations are associated with specific hand muscles, fingers, or finger movements [52, 53] and that the organization of such movements is somatotopically organized in the motor cortex [22], which is known to be somatotopically organized in a set of subregions that control different segments of the body [52]. More recent views suggest that movements are represented in motor areas as clusters of neurons coding for different action types or goals [54]. For instance, Graziano and Aflalo [55] demonstrated that electrical stimulation of the rostral precentral gyrus evokes coordinated movements of the hand and mouth and that these movements seem to be present even within the restricted repertoire of behaviors of infant primates. In general, preset motor repertoires for ethologically relevant actions have been demonstrated in the monkey cortex by mapping studies with microstimulation of motor cortical areas [56]. These results are consistent with recent neuroanatomical studies of the human brain, which have shown that representations of the hand and mouth in the human motor cortex are contiguous and show a high degree of overlap [22]. This organization is generally believed to produce adaptive movements by optimizing neural resources associated to effectors that are jointly involved in coordinated actions. For instance, we often close our hands to grab edible objects with the aim of bringing food to the mouth. At the cortical level, the grasping movement and the mouth opening movement are represented as motor synergies for which the closure of the hand is accompanied by the opening of the mouth. These hand/mouth movements are synchronous and coordinated to maximize their efficacy. It has been demonstrated that during electrical stimulation of the sensorimotor cortex, the mouth starts to open while the closing hand moves towards the face [22]. Furthermore, numerous kinematics studies by Gentilucci and colleagues show that the movement of the hand during grasping simultaneously affects the kinematics of the mouth during different motor tasks [23, 25, 26]. As a consequence, we have assumed that the synergistic activity of hand closing while smiling should facilitate the activation of the cortical areas connected to the mouth, facilitating the recruitment of the gracilis muscle without grinding of the teeth (synergistic activity therapy, SAT, Figure 1 [21]).

### 1.3. FIT-SAT at Home

**Figure 2:**
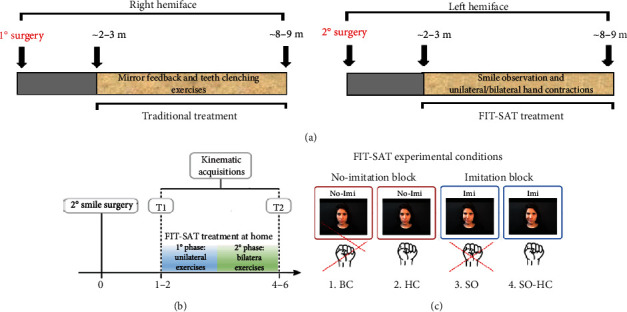
FIT-SAT treatment. (a) The FIT-SAT treatment was performed at home for about 6 months. After the first surgery, the right side of the face was rehabilitated by teeth clenching and mirror feedback. After the second surgery, the FIT-SAT treatment started as soon as the patient began to recruit the muscle. (b) The FIT-SAT treatment was divided into two phases: in the first (unilateral) phase, patients performed unilateral exercises in order to recruit the left transplanted muscle as soon as possible. The second (bilateral) phase started only after the patient was able to perform multiple repetitions of the unilateral left movement maintaining the posture for at least three seconds. From now on, the patient had to learn to coordinate the two sides of the face performing bilateral exercises. (c) Experimental condition: (1) no smile observation and no-hand contraction (baseline condition, BC), (2) no smile observation but hand contraction (HC), (3) smile observation but no-hand contraction (SO), and (4) smile observation and hand contraction (SO-HC).

The FIT-SAT treatment includes videos containing instructions and daily exercises to be performed at home for up to six months (Figure 2(a)). The protocol is divided into two phases. The first (unilateral) phase is aimed at increasing muscle strength with unilateral exercises avoiding teeth grinding and begins when the patient starts to recruit the transplanted muscle. This phase consists of a series of video clips of an actor performing only unilateral smiles which are then imitated by the patient. Each video clip contains instructions concerning both the coactivation of the hand closed as a fist and the specific number of repetitions that the MBS patient must perform each day. The duration of the first phase varies from patient to patient depending on the muscle recruitment. The second phase of the treatment begins only after the patient is able to perform multiple repetitions of the unilateral movement maintaining the posture for at least three seconds. The second (bilateral) phase is aimed at synchronizing the contraction of both sides in order to obtain a harmonious movement and a natural smile. This is achieved by presenting clips of an actor smiling bilaterally and by giving instruction about the coactivation of the hands. Bilateral exercises include modulation tasks in which the patient is asked to perform maximum and small (gentle) smiles^8^ in order to train and control the contraction force of the transplanted muscle/s.

One of the most complex aspects of home training is ensuring that patients perform the exercises correctly. To this aim, FIT-SAT's video clips start with instructions describing the exercises and during execution include auditory feedback in the form of an external voice that marks the timing of the observed smile to help the patient appreciate the rhythm of the smile to be performed. Thus, video clips help to sustain patient performance during home training. At each clinical assessment, patients are provided with clip materials according to their clinical status.

### 1.4. Assessing the Efficacy of FIT-SAT: Kinematic Acquisitions

**Figure 3:**
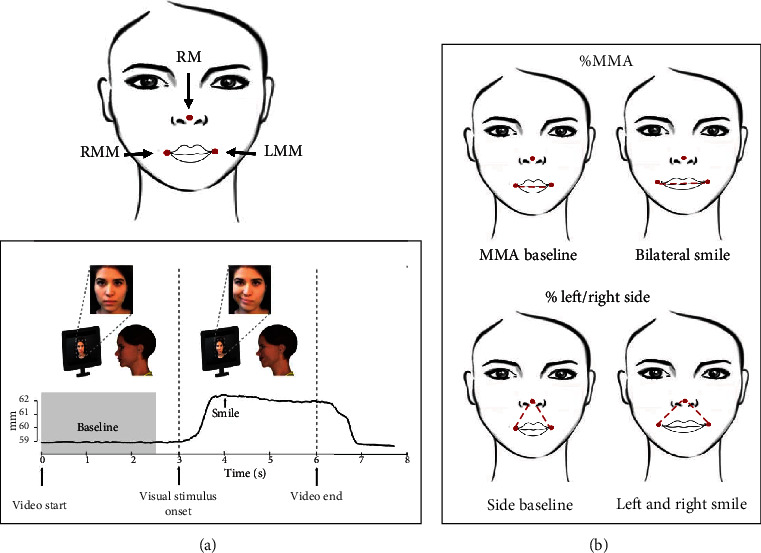
Kinematic parameters. (a) Example of one trial. The black line represents the excursion of the markers placed on the participant's mouth. The movement began after the participants observed the actress's smile and maintained the posture for about three seconds. The baseline is shown in gray. In this phase, the subject did not perform any movement. (b) Three reflective passive markers were placed on the participant's face (left mouth marker, LMM; right mouth marker, RMM; and reference marker, RM). Bilateral smile amplitude was calculated as the maximum Euclidian distance (MMA) in millimeters between the two lip corner markers (LMM and RMM). This measure was expressed as a percentage of the MMA at baseline (%MMA). Similarly, left/right side parameters were calculated as the Euclidian distances in millimeters between LMM or RMM lip corner marker and the nose marker (RM). Left/right side parameters were expressed as the percentage of side baseline (left or right, respectively, before movement onset).

The aim of the present study was to compare the efficacy of FIT-SAT with that of the traditional treatment. All patients underwent a two-stage surgery procedure (FFMT), spaced at least 9-12 months apart. They rehabilitated the right side of the face with traditional treatment [11, 15] first and about one year later the left side with FIT-SAT. We planned two kinematic acquisitions, one at the beginning of FIT-SAT (T1) and one at the end of treatment (T2, Figure 2(b)), to measure the three-dimensional motion of the patients' smile excursion. To compare the two treatments, we assessed maximal mouth aperture in the bilateral task between T1 and T2 to test how much the movement on one side of the face was the same as the movement on the other side. Specifically, we calculated the Euclidian distances between the left and right lip corner markers and the nose marker (Figure 3(b)). These parameters extrapolated from the bilateral smile provide an indirect measure of the left and right excursions, and their comparison may support the efficacy of the FIT-SAT treatment. Specifically, if the excursion of the left side at T2 was not different to that observed in the right side at T1, this would be evidence that FIT-SAT permitted a muscle recruitment as much as the traditional treatment [11]. Furthermore, we assess the efficacy of FIT-SAT to improve left muscle recruitment at the beginning of the treatment and to reduce asymmetry at the end of the treatment.

## 2. Material and Methods

### 2.1. Design and Participants

A small sample, pre- and postsurgery experimental design was adopted to study the efficacy of FIT-SAT. Four bilateral patients with MBS were included. Each patient was surgically treated from 2016 to September 2018 (right and left sides of the face, respectively) at the maxillofacial surgical unit at the University of Parma Hospital. Inclusion criteria were (1) a certified diagnosis of congenital and bilateral facial paralysis; (2) a transplanted segment of the gracilis muscle in both sides of the face and the motor nerve to the masseter muscle used for innervation; (3) recruitment of the right gracilis muscle subject to traditional treatment using teeth clenching; (4) recruitment of the left gracilis muscle subject to FIT-SAT treatment; (5) absence of congenital hand malformations; (6) absence of any psychiatric or physical illness at the time of participation; (7) age greater than 6 years.

All participants first underwent an operation on the right side of the face. For the rehabilitation of the right transplanted muscle, they underwent traditional treatment with teeth clenching (Pavese et al., 2016). After about one year, participants underwent a second surgery on the left side of the face. The patients underwent FIT-SAT treatment [21] after this second surgery (Table 1). Consequently, the first operated side (the right one) can be considered a “control side” as it represents activation of the gracilis muscle using traditional treatment. Clinical practice did not allow us to randomize the side subjected to the FIT-SAT. This can represent a potential limitation as facial expressions are more intensely expressed in the left side of the face [57], and previous works found a main effect of sidedness of the face on aesthetic judgments of pleasantness with the left hemiface usually more expressive [58]. However, for the purposes of this study, we were evaluating only the excursion of the smile and its symmetry while further studies will be needed to evaluate the expressiveness of the face.

Written consent was obtained after full explanation of the research procedure, in agreement with the Declaration of Helsinki. The treatment was approved by the Joint Ethics Committee of the Parma Department of Medicine and Surgery and of the Parma Hospital on 12nd October 2016 (Prot. 34819).

### 2.2. Procedure

When the left transplanted muscle innervated by the masseteric nerve gave signs of activation (approximately 2-3 months after the second surgery), the patients started FIT-SAT treatment at home and underwent the first kinematic acquisition (T1). The second kinematic acquisition (T2) occurred at the end of FIT-SAT (about 8-9 months after the second surgery) to measure the patients' progress in recruiting the transplanted muscle (Figure 2(a)).

Kinematic data were obtained by means of an optoelectronic system for motion analysis (SMART-DX-100 system, BTS Bioengineering). This system consists of four digital infrared cameras (with a frequency of 100 Hz), which detect the 3D movement of passive markers reflecting infrared rays emitted by illuminators with a spatial accuracy of at least 0.2 mm under the experimental conditions. Two markers were applied at the corners of the mouth (right and left mouth markers, RMM and LMM, respectively) and a further additional marker was placed on the nose (nose marker or reference point, RM, Figure 3(a)). Kinematic parameters were computed from each tracked trial using a custom program developed in RStudio 1.0.136 (https://www.rstudio.com/.).

Each kinematic acquisition consisted of 2 blocks: (1) imitation block in which an actress performed the smiles to be imitated by the patient; (2) no-imitation block in which an actress did not smile but provided the rhythm of the smiles during patients' assessment. Each block consisted of 40 repetitions of bilateral smiles and unilateral “half smiles.” After FFMT, patients have active movement excursion bilaterally, but they are able to move each side of their mouth independently. Thus, we asked the participants to perform a left half smile (unilateral task) to measure mouth excursion in the side rehabilitated with FIT-SAT. In both the imitation and no-imitation block, four experimental conditions were assessed:

1. *Smile observation and hand/s contraction (SO-HC)*: patients first observed a video clip in which an actress executed unilateral or bilateral smiles and then smiled while simultaneously closing their ipsilateral hand or both hands.
2. *Smile observation and no hand/s contraction (SO)*: patients observed/imitated unilateral or bilateral smiles maintaining their hand/s relaxed in a prone position
3. *No smile-observation and hand contraction (HC)*: the actor was visible on the screen and provided auditory feedback that marked the timing of the patients' smiles. Following the instructions of the actress on the video, the patients performed unilateral or bilateral smiles while simultaneously closing their ipsilateral hand or both hands.
4. *No smile observation and no hand contraction (BC)*: patients simply performed unilateral or bilateral smiles. We refer to this condition as the baseline condition (Figure 2(b)).

Patients performed 40 left and 40 bilateral smiles (10 repetitions for each experimental condition), 80 smiles in total. Each video lasted six seconds, three seconds of instruction followed by three seconds for performing the exercise (Figure 2(b)). Between each trial, patients could pause if they so desired. The order of the blocks was randomized among subjects.

### 2.3. Kinematic Parameters

Bilateral smile amplitude was calculated as the maximum Euclidian distance (MMA) in millimeters between the two lip corner markers (Figure 3(b)). This measure was expressed as a percentage of the MMA at baseline (%MMA), the MMA baseline corresponding to the Euclidian distance between the lip corner markers before movement onset (0 to 2.5 seconds, Figure 3(b)). For all trials, the %MMA was therefore calculated as follows:

(1) $$\begin{array}{c} \%MMA=\frac{MMA–MMA\;baseline}{MMA\;baseline}*100. \end{array}$$

In unilateral blocks (unilateral task), left %MMA was the Euclidian distance in millimeters between the two lip corner markers expressed as a percentage of the MMA at baseline.

Left (or right) smile excursions (left/right side) were also calculated as the Euclidian distances in millimeters between the left (right) lip corner marker and the nose marker (Figure 3(b)). Left/right side parameters were expressed as the percentage of side (% left/right side) with respect to the left/right side baseline (the Euclidian distance between the lip corner markers measured before movement onset (0 to 2.5 seconds, Figure 3(a))). For all trials, % left/right side was therefore calculated as follows:

(2) $$\begin{array}{c} \%Left\;side=\frac{Left\;side-Left\;side\;baseline}{Left\;side\;baseline\;}*100,\\ \%Right\;side=\frac{Right\;side–Right\;side\;baseline}{Right\;side\;baseline}*100. \end{array}$$

We also calculated the asymmetry index of the bilateral blocks (bilateral task) (%AI), which provides information to evaluate the attainment of a harmonious and natural movement. The AI was calculated with the following formula:

(3) $$\begin{array}{c} \%AI=\left(\frac{\max¯MMA-\min¯MMA}{\max¯MMA+\min¯MMA}\right)*100. \end{array}$$

A smile will be symmetrical as the value approaches 0% asymmetric as the value tends to 100% [59].

### 2.4. Statistical Analysis

The aim of this study was to compare the efficacy of standard treatment with FIT-SAT. Right and left sides of the face were operated in two phases (about one year apart). As a result, one side was rehabilitated before the other. All patients rehabilitated the right side of the face with traditional treatment [11] first and about one year later the left side with FIT-SAT. The main objectives were the following:

1. to assess the excursion of the left half smile (Left %MMA) among experimental conditions at T1
2. to assess an improvement in symmetry (%AI reduction) between T1 and T2
3. to compare participants' maximal mouth aperture between % right side at T1 and % left side at T2

We used linear mixed-effect models fit by maximum likelihood (LMM) to test the efficacy of the FIT-SAT treatment on the rehabilitation of the patients' smile. To select the best model that yields our data, we used the Akaike information criterion (AIC), which offers a principled balance between goodness-of-fit and model complexity [60]. The principal characteristic of this approach is the inclusion of random subject effects into regression models in order to account for the influence of subjects on their repeated observations. The information criteria (AIC values) together with log-likelihood statistics are reported and provide a way to assess the fit of a model based on its optimum log-likelihood value (Tables 2–4). Data analyses were performed using RStudio 1.3.1093 (https://www.rstudio.com/.) using the “Ime” function in the “nlme” package. The threshold for statistical significance was set at *p* < 0.05 for all analyses.

## 3. Results

### 3.1. Unilateral Task

To test the FIT-SAT conditions in facilitating the unilateral left excursion (first phase) at the beginning of the treatment, we entered left %MMA as the dependent variable and compared the fit of a generalized least squares (GLS) null model (m0__T1_, *y* ~ 1) with fixed intercept with that of a null model with random intercept (m1__T1_, *y* ~ (1 subjects)). m1__T1_ provided a superior fit than m0__T1_ (AIC_m0_T1_ = 700.4 and AIC_m1_T1_ = 589.9; *p* < 0.001). We then added the factor “condition” as a fixed effect to m1, generating m2__T1_ (*y* ~ condition + (1 subjects)). The comparison between models revealed that m2__T1_ provided a better fit (AIC_m2_T1_ = 581.3; *p* < 0.002, see Table 2).

**Figure 4:**
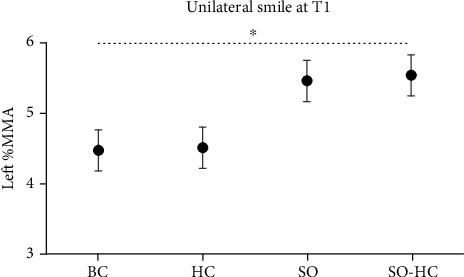
Results of unilateral task at T1. Left %MMA was the Euclidian distance in millimeters between the two lip corner markers expressed as a percentage of the MMA at baseline. All the experimental conditions are represented: smile observation followed by imitation of the same smile movement and ipsilateral hand contraction (SO-HC), smile observation followed by imitation of the same smile movement but without hand contraction (SO), no smile observation but hand contraction (HC), and no smile observation and no hand contraction (BC). Error bars represent SE (standard errors of the means).

Post hoc tests (Dunnett's) were performed to test the condition effects. We observed a significant increase in SO-HC (5.55 mm ± 0.4) in comparison to BC (4.47 mm ± 0.4, *p* = 0.005, Figure 4). No other comparisons were found to be significant (*p* > 0.05).

### 3.2. Bilateral Task

**Figure 5:**
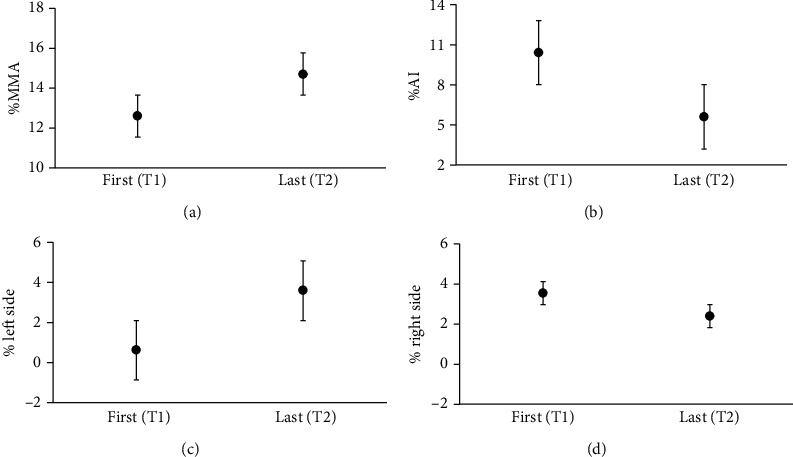
The graphs show the results of the bilateral analysis between the first (T1) and the last acquisition (T2). The parameters considered were (a) %MMA (the maximum Euclidian distance in millimeters between the two lip corner markers), (b) %AI (asymmetry index), (c) % left side (the Euclidian distances in millimeters between the left lip corner marker and the nose marker), and (d) right side (the Euclidian distances in millimeters between the right lip corner marker and the nose marker). Error bars represent SE (standard errors of the means).

On average, %MMA increased at the end of FIT-SAT treatment (T2) with respect to the beginning T1 (T1 = 12.59 mm ± 0.19, T2 = 14.66 mm ± 0.32; Figure 5(a)) whereas %AI decreased (T1 = 10.45 mm ± 0.52, T2 = 5.59 mm ± 0.24; Figure 5(b)). Similar to %MMA, in T2, the % left side increased in the percentage of excursion in comparison to T1 (T1 = 0.616 mm ± 0.11, T2 = 3.593 mm ± 0.24; Figure 5(c)) whereas the average values of the % right side show a slight decrease (T1 = 3.544 mm ± 0.28, T2 = 2.39 mm ± 0.15; Figure 5(d)).

**Figure 6:**
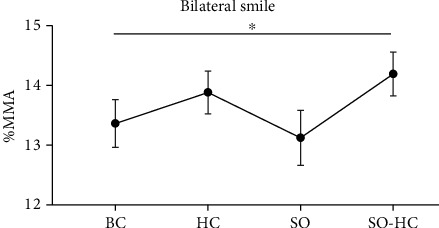
The graph shows the results of the bilateral task in both acquisitions considering the FIT-SAT conditions. Specifically, %MMA (the maximum Euclidian distance in millimeters between the two lip corner markers) increased in SO-HC (smile observation and hand contraction) with respect to the baseline (BC). Error bars represent SE (standard errors of the means).

We run LMM with a random intercept to account for the interindividual variability, and we compared models using the likelihood-ratio test. We entered all kinematic parameters as the dependent variables and compared the fit of a generalized least squares (GLS) null model (m0, *y* ~ 1) with fixed intercept with that of a null model with random intercept (m1, *y* ~ (1 subjects)). In %MMA, m1 provided a superior fit than m0 (AIC_m0_ = 1445.9 and AIC_m1_ = 1285.4; *p* < 0.001). We then added the factor “acquisition” as a fixed effect to m1, generating model 2 (m2, *y* ~ acquisition + (1 subjects)). The comparison between models revealed that m2 provided an even better fit (AIC_m2_ = 1234.8; *p* < 0.001), suggesting that mouth maximal aperture increased as a function of time. Finally, we added the factor “condition” as a fixed factor (m3, *y* ~ acquisition + condition + (1 subjects)) and interaction (m4, *y* ~ acquisition∗condition + (1 subjects)). The comparison between models revealed that m3 provided the better fit (AIC_m3_ = 1230.4; *p* < 0.015, see Table 3). Dunnett's comparisons were performed comparing each FIT-SAT condition (HC, SO, and SO-HC) with the control condition (BC). We observed a significant increase in SO-HC condition (14.19 mm ± 0.37) in comparison to BC (13.36 mm ± 0.39, *p* = 0.045, Figure 6). No other differences were found (*p* > 0.05).

We performed the same comparisons between models in the %AI variable. We observed a lower AIC values in both m0 vs. m1 and m1 vs. m2 comparisons (AIC_m0_ = 1595.7 and AIC_m1_ = 1509, *p* < 0.001; AIC_m2_ = 1402.9, *p* < 0.001; Table 3). Specifically, the factor “acquisition” improves the quality of the fit compared to m0 and m1 suggesting that, in T2 patients, smiles were more symmetrical than in T1 patients. Thus, the best explanation for the improvement in the quality of patients' smile was accounted by the factor “acquisition” which, in turn, reflects the effect of the FIT-SAT treatment over time (Figure 5(b)). Instead, model comparisons indicated that m3 and m4 did not improve the fitting (*p* > 0.05, Table 3).

To analyze the effect of the FIT-SAT treatment in activating the left muscle without teeth clenching, we further employed a LLM for left and right sides separately. Once again, the best model that yields our data in the excursion of % left side was accounted for by the acquisition factor (AIC_m2_ = 1121.8; *p* < 0.001, Figures 5(c) and 5(d)). The AIC values for each comparison between models are shown in Table 3.

### 3.3. Traditional vs. FIT-SAT Treatment Comparison

To examine the two treatments, we compared the % left side and % right side parameters at T1 and T2. The analysis procedure follows the previous one. The comparison between models revealed that m4 provided the better fit (AICm4 = 2238.3; *p* = 0.001, see Table 4).

**Figure 7:**
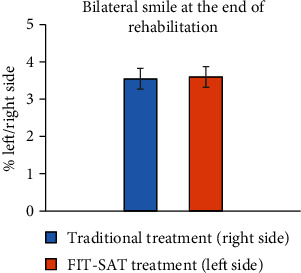
The graph shows the results between the % right side and the % left side (the Euclidian distances in millimeters between the left/right lip corner marker and the nose marker, Figure 3(b)) at the first acquisition (T1, blue) and at the last acquisition (T2, orange), respectively. Error bars represent SE (standard errors of the means).

Dunnett's comparisons were performed comparing % right side T1 with the other conditions. We found a significant difference between % right side T1 and % left side T1 (3.54 ± 0.28 and 0.62 ± 0.11, respectively; *p* < 0.001, Figure 7) and % right side T2 (1.17 ± 0.21, *p* < 0.001). No difference was found between % right side T1 and % left side T2 (*p* > 0.05).

## 4. Discussion

Peripheral facial palsy, involving a lesion of cranial nerves involved in facial mimicry, is typically correlated to important functional and aesthetic deficits. Patients with congenital unilateral or bilateral facial palsy show reduced or absent expressivity; they either cannot smile (when affected bilaterally) or find it very difficult to smile (unilateral paralysis). In addition, they cannot grimace or close their eyes normally. Finally, because of the lack of strength in their lip muscles, they also have problems with chewing, swallowing, and speaking. Surgical interventions are aimed at reducing the symptoms and restoring a degree of facial mobility (i.e., facial reanimation [7]). Despite the strong negative impact of facial palsy on psychosocial functioning and quality of life [61], however, current approaches to postsurgery treatment remain largely unsatisfactory. Following muscle transplant, traditional rehabilitation programs are aimed at activating newly formed motor circuit**s** under the control of the masseteric nerve. Thus, patients are initially encouraged to practice biting in front of a mirror [11]. However, the practice of teeth clenching, although extremely effective in recruiting the transplanted muscles [12], leads to difficulties in separating chewing from smiling and remains divorced from mimicry processes, which play an important part in social interactions. As an additional problem, clinicians report poor compliance with prescriptions involving home training under mirror feedback, presumably due to the negative consequences of facial palsy for self-perception [14].

Here, we tested a new neurorehabilitative protocol (FIT-SAT) that exploits the properties of the mirror system as well as hand-mouth synergies [22, 55] related to the somatotopic organization of the motor cortex. Our results support the feasibility of FIT-SAT as an alternative to mirror feedback therapy. Specifically, we analyzed the excursion of the lips in four patients with bilateral paralysis. The patients rehabilitated the right side of the face with the traditional treatment involving teeth clenching [11], whereas they rehabilitated the left side with FIT-SAT [21]. Using 3D kinematic acquisitions, the recruitment of the left transplanted muscle was monitored by the second intervention onwards. A beneficial effect of the SO-HC condition was observed in the unilateral task at the first acquisition. Specifically, smile observation (SO) associated to hand contraction (HC) was effective in recruiting the transplanted muscle in the early phase of the treatment (unilateral phase) resulting in a greater left side excursion with respect to the baseline (BC).

The unilateral phase of the FIT-SAT treatment finished when patients were able to recruit the transplanted muscle even in the absence of hand contraction. Once the first unilateral phase was completed and the muscle of the left side had been fully recruited, the second bilateral phase began. This second phase was aimed at synchronizing the contraction of both sides in order to obtain a harmonious movement and a natural smile. The most important result observed in the bilateral task was the improvement in smile symmetry at the end of the treatment.

In the bilateral task, we also observed a condition effect. Specifically, results showed an increase in the maximal mouth aperture in SO-HC in comparison to BC suggesting that the hand (effective in early muscle recruitment) was still useful at the end of the treatment by increasing lip excursion during smiling when associated with smile observation. Nevertheless, it should be noted that the maximal mouth aperture is not the parameter that can best describe an improvement in the smile quality, and a greater maximal mouth aperture does not necessarily imply that the patient achieved a more harmonious and natural smile. As an example, an excessive excursion might rather indicate poor quality of modulatory control of muscles.

Finally, in the bilateral task, we did not find significant differences comparing the excursion of the right side at T1 (side of the face rehabilitated with traditional treatment) and the left side at T2 (side of the face rehabilitated with FIT-SAT treatment). This last result supports the conclusion that FIT-SAT treatment may be as effective as the traditional treatment in recruiting muscles involved in smiling after smile surgery. Notably, we found a significant decrease in right side excursion between T1 and T2. This effect could depend on FIT-SAT treatment. In fact, in the second phase of the FIT-SAT, bilateral exercises of modulation were included. This may have resulted in better smile control making the subject aware of the force of muscle contraction. These results, although promising, will require further investigations; in particular, it will be interesting to verify the modulatory effects of the FIT-SAT treatment over time.

One of the foremost goals for MBS patients undergoing postsurgical rehabilitation is to achieve a smile that is as harmonious and natural as possible. Our results indicate that FIT-SAT may be helpful in this respect as well, as we observed that smile symmetry improved between the first and last acquisitions. Thus, the combined use of smile observation, smile reproduction, and contingent hand contraction resulted in a reduction of the anomalous asymmetry.

A final consideration is in order in relation to the social function of smiling. The absence of a spontaneous smile is what brings most problems to patients suffering from facial paralysis since it impairs communication and social interaction [62]. In these patients, smile production cannot be controlled by a sensitive nerve, which means that they must control the smile consciously. Nevertheless, some authors have reported that, over time, some MBS patients develop an ability to activate their smile in social situations, especially if they underwent smile surgery at an early age [63, 64]. These reports have been used to propose that greater brain plasticity in younger patients leads to the achievement of a spontaneous smile after neural reorganization of involved motor processes [63, 64]. We speculate that FIT-SAT could favor this process. The motor and premotor cortexes have been demonstrated to be part of a visuomotor coupling mechanism (i.e., the mirror neuron system [65]). During the observation of an action/gesture, our motor system resonates with that of the model because the observer is automatically recruiting the same motor programs of the model. Motor resonance mediated by the above-mentioned sensorimotor mirror system could support basic functions such as action perception, understanding, and imitation of the observed agent [66], including mimicry which normally occurs during face-to-face interactions [67].

Here, both SO and SO-HC conditions exploit the principles of AO [16] to facilitate the recruitment of the transplanted muscle. Specifically, two mechanisms intervene: one is linked to the voluntary production of the smile, involving motor areas that provide awareness to the movement; the other one is based on activities of the MNS, an observation-execution matching system activated both during the execution of a motor act and during the passive observation of other people performing the same movement [29, 39]. In other terms, we map what we observe onto our own neural motor representations for a specific action, sensation, or emotion [1, 47, 48]. In fact, MNS is thought to crucially subserve emotion recognition processes. Not by chance, the temporary reversible lesion of the MNS (due to repetitive transcranial magnetic stimulation) is associated with performance deficits on tasks requiring the recognition of facial expressions of emotion [68]. To date, how voluntary and automatic processes interact is not entirely clear. Investigations conducted by Caruana et al. [69] by means of electrical stimulation during brain surgery supported the role of frontal operculum (FO) in both observation and the voluntary control of facial expressions. Its stimulation in patients that underwent brain surgery induced the production of a smile. Moreover, previous brain imaging studies have reported the activation of the FO both during the voluntary imitation and during the passive observation of a smile [70–72]. Thus, thanks to its connectivity pattern with other brain structures involved in emotion processing, FO would result in a sort of “gate” between the voluntary motor system and the emotional network and crucially subserving facial expression production and recognition in the context of social interactions. Thus, FIT-SAT may improve not only the recovery of motor function but also the spontaneity of the smile normally occurring in everyday social situations. In fact, when the patient smiles at another person who responds with eye contact [73, 74] and by smiling back, there is a powerful reinforcement both consciously and unconsciously, which likely aids the learning process as the patient can realize that the movement was indeed recognized as a smile. Such a speculation is supported by studies on mother-infant interactions, showing that infants tend to increase social expressiveness when their mothers mirror their facial expressions [75]. Moreover, such mother mirroring has an impact on the development of cortical motor circuits involved in facial expression perception [76]. However, at the moment, we have no actual evidence of the efficacy of FIT-SAT treatment in the production of a spontaneous smile, and future follow-up studies are needed to investigate the validity of this hypothesis.

### 4.1. Limitations of the Study

Because of the rarity of the syndrome, we could only include a small number of participants, and this precludes generalization of our results. For future studies, the research question should be addressed in a larger sample. For reasons related to clinical practice, it was not possible to randomize the side of the face rehabilitated with the FIT-SAT. Future studies will need to consider this aspect in order to obviate possible effects caused by hemispheric lateralization in emotion processing [58].

## 5. Conclusion

Our results indicate that hand contraction and smile observation may be as efficacious as traditional teeth clenching treatment, while bypassing patients' difficulties in working with the mirror and allowing a correct dissociation between chewing and smiling. To the best of our knowledge, this study is the first to apply an AOT-based rehabilitation approach [17, 18, 77] to patients with facial paralysis who undergo smile surgery [7, 78] and to integrate knowledge derived from neuroscience such as hand-mouth synergy with the clinical rehabilitation needs of these patients [22, 23, 43, 54]. Although this preliminary data is encouraging, further confirmation will be necessary with a greater number of patients and with experimental designs including assessments of FIT-SAT after the first muscle transplant.

## Figures and Tables

**Table 1:** Patient classification: demographics and clinical characteristics of patients.

ID_num	Sex	Age	Patients classification	Type of paralysis	Type of smile surgery	Transplanted muscle	1° smile surgery	2° smile surgery	FIT-SAT duration
MBS01	f	11	Bilateral Moebius	Complete bilateral paralysis	Free muscle transfer	Right side: gracile	Right side	Left side	235
						Left side: gracile	12-05-2015	21-01-2016	
MBS02	f	40	Bilateral Moebius	Complete bilateral paralysis	Free muscle transfer	Right side: gracile	Right side	Left side	205
						Left side: gracile	03-02-2016	21-04-2017	
MBS03	f	7	Bilateral Moebius	Complete bilateral paralysis		Right side: gracile	Right side	Left side	167
					Free muscle transfer	Left side: gracile	11-06-2016	31-08-2017	
MBS04	m	8	Bilateral Moebius	Complete bilateral paralysis	Free muscle transfer	Right side: gracile	Right side	Left side	147
						Left side: gracile	01-07-2015	18-01-2017	

**Table 2:** FIT-SAT treatment efficiency: best fit mixed-effect models (unilateral smile in T1).

Parameters	Model	df	AIC	BIC	LogLik	Test	L. ratio	*p* value
Left %MMA	m0__T1_	2	700.4	706.5	-348.2			
	m1__T1_	3	589.9	598.9	-291.9	m0__T1_ vs. m1__T1_	112.6	<0.001
	m2__T1_	4	581.3	599.4	-284.8	m1__T1_ vs. m2__T1_	14.5	<0.002

**Table 3:** FIT-SAT treatment efficacy: best fit mixed-effect models. Information of the mixed-effect models used for different kinematic parameters.

Parameters	Model	df	AIC	BIC	LogLik	Test	L. ratio	*p* value
%MMA	m0	2	1445.9	1453.2	-721.0			
	m1	3	1285.4	1296.3	-639.7	m0 vs. m1	162.5	<0.001
	m2	4	1234.9	1249.4	-613.4	m1 vs. m2	52.5	<0.001
	m3	7	1230.4	1255.8	-608.2	m2 vs. m3	10.5	0.015
	m4	10	1229.3	1265.5	-604.6	m3 vs. m4	7.2	0.066

%AI	m0	2	1595.7	1602.9	-795.9			
	m1	3	1509.1	1519.8	-751.5	m0 vs. m1	88.7	<0.001
	m2	4	1402.9	1417.2	-697.5	m1 vs. m2	108.2	<0.001
	m3	7	1408.0	1432.9	-697.0	m2 vs. m3	1.0	0.813
	m4	10	1413.2	1448.9	-696.6	m3 vs. m4	0.8	0.859

% left side	m0	2	1337.5	1344.7	-666.7			
	m1	3	1267.1	1277.9	-630.5	m0 vs. m1	72.4	<0.001
	m2	4	1121.8	1136.3	-556.9	m1 vs. m2	147.2	<0.001
	m3	7	1125.9	1151.3	-556.0	m2 vs. m3	1.9	0.598
	m4	10	1130.8	1167.0	-555.4	m3 vs. m4	1.2	0.764

% right side	m0	2	1331.0	1338.2	-663.5			
	m1	3	1123.3	1134.2	-558.7	m0 vs. m1	209.7	<0.001
	m2	4	1093.2	1107.7	-542.6	m1 vs. m2	32.2	<0.001
	m3	7	1091.4	1116.7	-538.7	m2 vs. m3	7.8	0.050
	m4	10	1093.1	1129.4	-536.6	m3 vs. m4	4.2	0.238

**Table 4:** FIT-SAT treatment efficiency: best fit mixed-effect models (bilateral smile, % left vs. right side).

Parameters	Model	df	AIC	BIC	LogLik	Test	L. ratio	*p* value
% left/right side	m0	2	2678.1	2686.7	-1337.0			
	m1	3	2440.9	2453.8	-1217.4	m0 vs. m1	239.2	<0.0001
	m2	4	2418.3	2435.6	-1205.2	m1 vs. m2	24.6	<0.0001
	m3	7	2397.6	2419.2	-1193.8	m2 vs. m3	22.7	<0.0001
	m4	10	2238.3	2264.2	-1113.1	m3 vs. m4	161.3	<0.0001

## Data Availability

Data are available upon request.

## Abbreviations

MBS: Moebius syndrome
FFMT: Free functional muscle transfer
FIT: Facial imitation treatment
SAT: Synergistic activity therapy
AOT: Action observation therapy.

## Supplementary Materials

Supplementary Materials Free functional muscle transfer (FFMT) description.
