# Supplementary material for: A New Neurorehabilitative Postsurgery Intervention for Facial Palsy Based on Smile Observation and Hand-Mouth Motor Synergies
Source: Neural Plast. 2021 Mar 24;2021:8890541. doi: 10.1155/2021/8890541 (PMC8016575; doi:10.1155/2021/8890541)
Supplement: Supplementary Materials — Free functional muscle transfer (FFMT) description. [file 8890541.f1.docx]

**Free functional muscle transfer, FFMT**

The FFMT is a surgery technique which takes a muscle from elsewhere in the patient's body (usually the gracilis muscle from the leg) and grafts it onto the corners of his mouth. In parallel to this the motor nerve to the masseter muscle is used as the donor nerve. Such surgical procedure is performed in order to give to the patients a mechanical smile ability. Each side of the face is treated in separate stages and the average time taken to complete the treatment is around two years^1^. Typically, the first surgery is performed on the right side when the child has turned 6-7 years. It should ideally be completed before the child becomes fully aware of his/her palsy and the psychological implications begin to take hold^1,2^, even though at this age there are already psychological negative consequences due to social exclusion and stigmatization among peers.

In unilateral palsy, the best donor nerve for restoring the smile is the contralateral facial nerve which permits synchronous and spontaneous activity of the transplanted muscle^3^. Unfortunately, in patients with MBS, bilateral facial nerve palsies preclude cross-face nerve grafts for facial reanimation. Thus, the masseteric nerve offers an alternative to innervate the functional free gracilis transfer due to its consistent anatomy, close proximity to the transplanted muscle, strong motor impulse and relative lack of donor morbidity^4–6^. The procedure involves transfer a segment of the gracilis muscle to the face and attachment to the masseteric nerve (the trigeminal motor branch of the 5th cranial nerve), which controls the muscles for chewing^1^ creating a new smile circuit in which the muscles contraction is determined by teeth clenching. The use of the masseteric nerve provides a strong smile and the procedure results in first contractions after approximately 4 to 6 months when the growth of the nerve has reached the transplanted muscle with the generation of electrical pulses to the transplanted muscles. Nevertheless, the use of the masseteric nerve results in a not completely spontaneous smile and only with practice through a prolonged period of rehabilitation, the majority of patients is capable to produce a voluntary and symmetric smile. In fact, the trigeminal nerve is responsible for sensation in the face and for the motor control of movements, such as biting and chewing, and it cannot replace the spontaneous movements typically recruited during emotional arousal and social interactions. Consequently, after the surgeries, patients have significant difficulties in achieving the capacity to spontaneously smile, in particular without producing significant motor asymmetries.

**Reference**

1. Bianchi B, Copelli C, Ferrari S, Ferri A, Sesenna E. Facial animation in patients with Moebius and Moebius-like syndromes. *Int J Oral Maxillofac Surg*. 2010;39(11):1066-1073. doi:10.1016/j.ijom.2010.06.020

2. Leckenby J, Grobbelaar A. Smile restoration for permanent facial paralysis. *Arch Plast Surg*. 2013;40(5):633-638. doi:10.5999/aps.2013.40.5.633

3. Bianchi B, Ferri A, Poddi V, et al. Masseteric nerve for gracilis muscle re-innervation in unilateral facial palsy: Impact on quality of life. *J Craniomaxillofac Surg*. 2017;45(7):1051-1057. doi:10.1016/j.jcms.2017.03.021

4. Bianchi B, Ferri A, Ferrari S, et al. Cross-facial nerve graft and masseteric nerve cooptation for one-stage facial reanimation: principles, indications, and surgical procedure. *Head Neck*. 2014;36(2):235-240. doi:10.1002/hed.23300

5. Borschel GH, Kawamura DH, Kasukurthi R, Hunter DA, Zuker RM, Woo AS. The motor nerve to the masseter muscle: an anatomic and histomorphometric study to facilitate its use in facial reanimation. *J Plast Reconstr Aesthet Surg*. 2012;65(3):363-366. doi:10.1016/j.bjps.2011.09.026

6. Coyle M, Godden A, Brennan PA, et al. Dynamic reanimation for facial palsy: an overview. *Br J Oral Maxillofac Surg*. 2013;51(8):679-683. doi:10.1016/j.bjoms.2012.12.007
